# High-intensity laser application in Orthodontics

**DOI:** 10.1590/2177-6709.22.6.099-109.sar

**Published:** 2017

**Authors:** Eduardo Franzotti Sant’Anna, Mônica Tirre de Souza Araújo, Lincoln Issamu Nojima, Amanda Carneiro da Cunha, Bruno Lopes da Silveira, Mariana Marquezan

**Affiliations:** 1 Universidade Federal do Rio de Janeiro, Departamento de Odontopediatria e Ortodontia (Rio de Janeiro/RJ, Brazil).; 2 Universidade Federal de Santa Maria, Departamento de Odontologia Restauradora (Santa Maria/RS, Brazil).; 3 Universidade Federal de Santa Maria, Departamento de Estomatologia, Disciplina de Ortodontia (Santa Maria/RS, Brazil).​

**Keywords:** Laser therapy, Orthodontics, Oral surgery

## Abstract

**Introduction:**

In dental practice, low-level laser therapy (LLLT) and high-intensity laser therapy (HILT) are mainly used for dental surgery and biostimulation therapy. Within the Orthodontic specialty, while LLLT has been widely used to treat pain associated with orthodontic movement, accelerate bone regeneration after rapid maxillary expansion, and enhance orthodontic tooth movement, HILT, in turn, has been seen as an alternative for addressing soft tissue complications associated to orthodontic treatment.

**Objective:**

The aim of this study is to discuss HILT applications in orthodontic treatment.

**Methods:**

This study describes the use of HILT in surgical treatments such as gingivectomy, ulotomy, ulectomy, fiberotomy, labial and lingual frenectomies, as well as hard tissue and other dental restorative materials applications.

**Conclusion:**

Despite the many applications for lasers in Orthodontics, they are still underused by Brazilian practitioners. However, it is quite likely that this demand will increase over the next years - following the trend in the USA, where laser therapies are more widely used.

## INTRODUCTION

The term LASER is an acronym for Light Amplification by Stimulated Emission of Radiation. The first effective laser was developed in the 1960s, although the theoretical framework had been laid in the early 20^th^ century. Since then, lasers have been widely used in several routine applications, as laser pointers, barcode reading devices, CD/DVD/Blu-ray readers, scanners, firearms sights, fiber-optic communication, visual aids and graphic design for the cinema industry, and ultimately in health care, for Medical, Physical Therapy, and Dental fields.

In Dentistry, lasers are used in two major applications: biostimulation and surgery. The lasers applied for biostimulation procedures - in other words, for the activation of regenerative and healing processes - are the so called low-level laser therapy (LLLT) and operate under 500 mW. For this purpose, the diode and helium neon (HeNe) lasers stand out, depending on their active medium. Lasers that work beyond the 500 mW range are applied for high-intensity laser therapies (HILT), also called surgical lasers, given their tissue cutting capacity. For such uses, the CO_2_, Nd:YAG, Erbium (Er:YAG, and Er,Cr:YSGG) and diode lasers are the main examples. 

In Orthodontics, LLLT has been applied to relieve pain associated with orthodontic movements,^1^ ^-^ ^3^ accelerate bone regeneration after rapid maxillary expansion,^4^ ^-^ ^6^ and enhance orthodontic tooth movement.^7^ ^-^ ^12^ Although there are several protocols for using LLLT, its effectiveness is determined by the frequency of applications in-between the activation appointments, which makes it less attractive as a routine procedure.

HILT, on the other hand, has become increasingly popular among orthodontists, especially in the USA. It is used for quickly and effectively addressing soft tissue complications associated to orthodontic treatment^13^ through bloodless and atraumatic surgical interventions. The benefits of using HILT for soft tissue oral surgery include better hemostasis, decreased postoperative pain and infection rate, minimal tissue contraction, little or no need for sutures, shorter surgical stages, decreased trauma, edema and scarring,^14^ ^-^ ^20^ besides the reduced need for local anesthetics.

Among the high-intensity laser therapies to be used in orthodontics, the diode lasers play a special role given its superficial cutting ability, providing safer procedures after its shallow penetration and reduced risks of causing pulp damage. Besides, HILT devices tend to be more portable and cost-effective.^21^ Its wavelength ranges between 810 and 1,064 nm^21^ and is absorbed by pigmented tissues, that contain haemoglobin, melanin, and collagen. Provided recommended protocols are observed, they have no impact on dental or bone tissues for they have greater affinity for soft tissues.^22^ The laser/tissue interaction may lead to coagulation, denaturation of proteins, vaporization, and carbonization in the affected areas depending on the amount of energy emitted. This process seals the blood vessels promoting hemostasis, inhibits the pain receptors on the incision area, lowers the risk of infection, and enhances healing.^22^

This study will explore HILT indications for addressing soft tissue problems associated with orthodontic treatment as well as other hard tissue and dental materials applications. 

## HILT INDICATIONS IN ORTHODONTICS

The main indication for HILT in orthodontics is in soft tissue surgeries such as gingivectomy, ulotomy, ulectomy, frenectomies, and fiberotomy. Before delving into each of them, some shared features of surgical procedures shall be presented.

**Figure 1:**
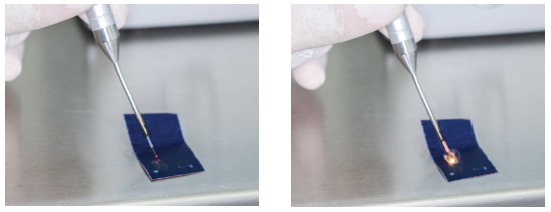
Activation of the laser tip with articulating paper.

Having in mind that diode lasers emit light to tissues through optical fiber cables or disposable optical fiber tips, the first step is to prepare this part of the instrument. For laser devices that use optical fiber cables, clinicians should properly remove the external coating to expose the internal glass fiber unit before using it. Before every single patient session, 2 to 3 mm of the fiber should be cut off/removed to prevent cross-contamination. Having done that, the tip should be “activated” by applying some sort of pigment on it, as to concentrate energy on that area - articulating paper can be used for that purpose (Fig 1). For disposable optical fiber tips, there is no need to cut off the tip, but the tip activation step is still required.^13^

Following this first step, laser intensity settings must be adjusted. It is advisable to use as low power as convenient to a given procedure, in order to avoid thermal damage to surrounding tissues.^13^ Most soft tissue procedures can be performed at a 1 to 1.2 W setting. In higher density soft tissues areas, like the palate or distally to lower molars, settings as high as 1.4 W may be required, while frenectomies usually require settings of around 1.6 W.^13^

Once power set up is complete, the energy delivery mode should be selected: continuous or pulsed waves. Whenever working with a diode laser, continuous waves should be set for most ablation procedures.^13^

Next step is the patient’s anesthesia. In some cases, the anesthetic gel is enough.^13^ After drying out the soft tissue area, the topical anesthetic gel is rubbed over the area with a cotton swab for 3 to 4 minutes. For higher-density tissue areas like the palate, distally to erupting molars, and at the frenulum, only the anesthetic gel may not be enough and injected local anesthesia is required.^13^ Practitioners should assess both the anesthetic effect and the patient’s sensitivity to decide if more local anesthesia is required.

The surgical procedure *per se* starts when the surgeon moves the end of the laser tip with a slow and careful “paint brushing” motion, making contact with the target tissue. Special attention should be taken towards avoiding to keep the laser tip for too long over a specific area, so as not to carbonize or unnecessarily damage the tissue. For ablation procedures, the use of the aspirator is paramount to remove vapor fumes, that may contain bacteria and unpleasant odors. It is also recommended to use a wet gauze^13^ to wipe off the tissue build ups that eventually start to accumulate at the tip of the instrument. 

While no specific postoperative care is required, patients should still be advised to keep the treated area clean and free of biofilm. Special care should be taken as to avoid extremely hot and cold foods that may cause pain or other complications. Ordinary pain killers can be prescribed after more complex surgical procedures.^13^

### Gingivectomy

**Figure 2:**
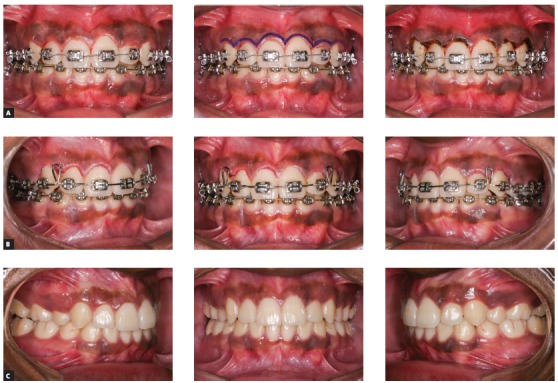
Intraoral photos showing a gingivectomy surgery during orthodontic finishing stage. A) Pre-operative aspect, marking the limits for tissue removal, and postoperative aspect. B) 24 hours after the surgery. C) Final aspect after bracket removal.

Patients undergoing orthodontic treatment with fixed appliances may develop gingival hyperplasia, mostly caused by chronic inflammatory processes.^23^ ^-^ ^25^ In such cases, gingival hyperplasia can be treated with gingivectomy together with improved mechanical plaque control, as well as with better overall oral hygiene by the patient.^23^ ^-^ ^25^ Gingivectomies can also be of great help for bonding the braces in patients with short clinical crowns as well as improving the aesthetics after the orthodontic treatment (Fig 2).

Regardless of whether the gingivectomy is to be performed with a scalpel or HILT, periodontal surgery principles and the concept of biological width should be abided by at all times. Biological width is around 3 mm, comprised of 1 mm junctional epithelium, 1 mm supercrestal attached connective tissue and 1 mm gingival sulcus. If this biological width is invaded by an excessive removal of gum tissue after gingivectomy, this tissue may grow back again because the biological width tends to return to its original dimensions. Chronic inflammation and unpredictable bone loss are commonly seen when the limits of the restoration exceed the biological width after surgery. With that in mind, cases that require significant tissue removal deliver better results whenever treated with crown lengthening procedures. The gingivectomy involves anesthesia and periodontal probing, in order to assess the tissue that will be safely removed around the teeth. This probing can be used as a reference to point out the amount of tissue to be removed.

The laser power should initially be set at 1 to 1.2 W.^13^ The optical fiber should be positioned towards the gingival tissue perpendicularly to the clinical crown and the removal of redundant tissue should start from the proximal aspect. A saline embedded gauze should be used to clean the area and remove remnants of gingival tissue. After applying the laser, the gingival margin should be assessed and refined with chisels or a 15C scalpel, if needed.

Laser-aided gingivectomy procedures do not require the use of surgical dressing, and the prescription of pain killers depends on patients’ tolerance. Another important aspect to be assessed is the post surgical tissue healing since the contraction of the margins may yield poor functional and aesthetic results after scar tissue is formed. A previous study found that the use of CO_2_ laser to treat gingival hyperplasia during orthodontic treatment had led to increased clinical crowns only during the immediate postoperative. However, between 30 to 60 days after surgery there were no significant changes to the gingival sulcus depth, probably caused by the gingival margins relapsing into physiological conditions.^25^

### Ulotomies and ulectomies

**Figure 3:**
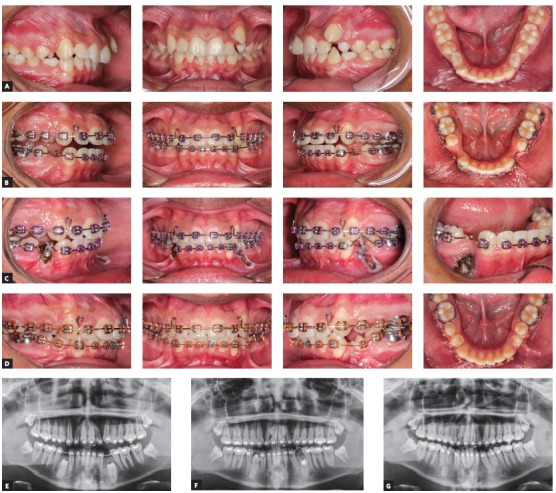
Intraoral photos showing exposure of first lower premolars for orthodontic traction. A) Clinical case: initial photo. B) Space distribution and preparation of the force system for tractioning teeth #34 and #44. C) Postoperative status immediately after surgery: Note that the amount of soft tissue removed during the exposure of #44 was more conservative, in comparison to the left side due to the apical positioning of #34, at the alveolar mucosa level. D) 12 months after exposure. E, F) Panoramic radiographs before and after traction. G) Radiograph showing the dental alignment and leveling of teeth #34 and #44.

**Figure 4:**
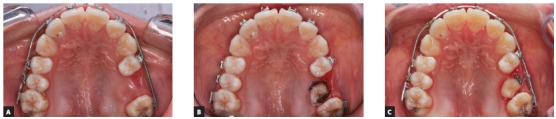
Intraoral occlusal photos showing the exposure of the upper left second premolar for orthodontic movement: A) pre-operative aspect, B) postoperative aspect, C) healing of tissue observed during the movement of the tooth.

**Figure 5:**
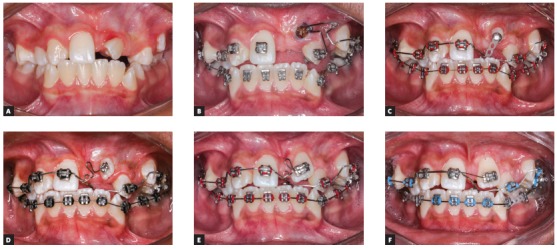
Intraoral photos showing the exposure of the upper left central incisor for orthodontic movement: A) initial clinical case, B) Post-surgery status. C-F) Tissue healing observed during the movement of tooth #21.

The surgical exposure of fully impacted or partially erupted teeth is performed as to allow the bonding of orthodontic devices on tooth surface and is another application of HILT in Orthodontics (Figs 3, 4, and 5).

An attentive assessment of the soft tissue to be excised must be carried out in anticipation. Ulotomies and ulectomies have good results when the tissue is keratinized. The surgical exposure of impacted teeth through non keratinized or unattached gingiva may result in loss of attached gingiva when those teeth are brought into the arch line through orthodontic forces.^13^

The laser intensity should be set to 1 to 1.2 W. When applied to areas with higher soft tissue density, like the palate or distally to lower molars, a power setting as high as 1.4 W^13^ may be required.

### Labial frenectomy

The labial frenum is a membrane that stretches along the midline from the internal surface of the lip to the alveolar mucosa. Among other functions, it limits lip movement, stabilizes the midline, and prevents unnecessary exposure of both alveolvar mucosa and gingiva. In newborns, the upper labial frenum extends all the way to the incisive papilla and takes part in suckling function. As teeth start to develop, the frenum goes through a gradual atrophy and attaches to the alveolar mucosa. However, in some cases the upper labial frenum keeps attached to the incisive papilla (low frenum attachment or frenum hypertrophy). A low frenum may cause interincisal diastemas. The diagnosis of this condition can be done by observing wether stretching the lip leads to an ischemic incisive papilla. A differential diagnosis will rule out mesiodens (X-ray), habits, absence of lateral incisors or hereditary factors.^26^ Although less frequently, hypertrophic lower labial frenum may also cause diastemas,^27^ but leading instead to another common problem: gingival recessions that may occur when the frenum is attached too close to the marginal gingiva.^13^

The correlation between the upper lip frenum and interincisal diastema has caused upper labial frenectomies to be performed on a regular basis as a preventive procedure until the mid-1940s.^28^ Not long after that, clinicians started to realize that there was a tendency for attachment to gradually migrate from the palatal to the buccal aspect throughout life as a consequence of the alveolar growth and the eruption of incisor teeth. Therefore, the current recommendation is to wait until the permanent canines erupt before proceeding to a frenectomy.

**Figure 6:**
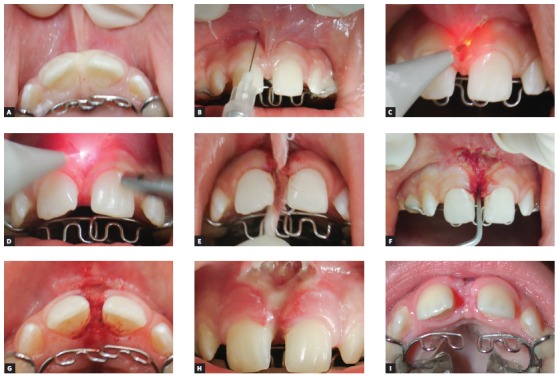
Intraoral photos showing a labial frenectomy case. A) Traction of upper lip. B) Local injectable anesthesia. C, D) HILT-aided frenum detachment. E, F) Mechanical removal of periodontal fibers. G) Immediate postoperative aspect. H) 24-hour postoperative. I) One month follow-up. It is important to note that LLLT was performed on a weekly basis, to accelerate the surgical wound healing process.

Regarding HILT surgical technique, after anesthesia and laser preparation stages, the lip should be stretched as to allow for an anatomical assessment of the frenum. Laser irradiation starts from the central part of the frenum towards the sulcus until the redundant frenum tissue is removed (Fig 6). Right then, it is noticed how fibers are removed under no bleeding. The recommended laser setting is 1.6 W.^13^ When frenum fibers are deeply inserted, detaching periodontal instruments might be used to excise them. Laser therapy should not be used adjacently to bone tissues given the risk of thermal damage and tissue necrosis. No suturing is required since a secondary intention healing will take place.

### Lingual frenectomy

**Figure 7:**
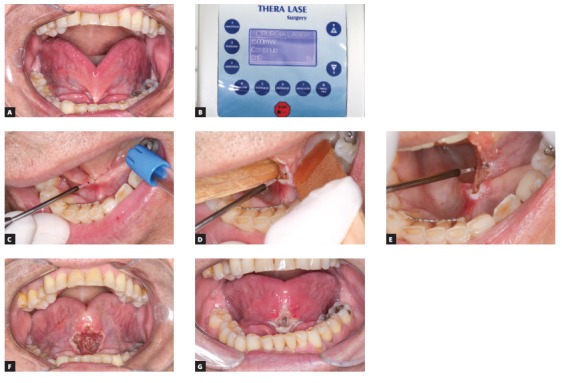
Lingual frenectomy clinical case. A) Pre-operative aspect. B) Laser device set up. C, D, and E) Frenum detachment. F) Immediate postoperative aspect. G) One week follow-up. Note the improvement in the range of movements and the shape of the tip of the tongue.

Ankyloglossia is the abnormal development of the tongue, characterized by the presence of a short and tight lingual frenum that limits tongue movements. It may lead to swallowing and speech disabilities, malocclusion as well as potential periodontal problems.^13^ A visual diagnosis can be done by asking the patient to elevate the tongue to the palate, making good evidence of the typical heart-shaped tongue with movement limitations (Fig 7A). The lingual frenum ablation can be performed in patients of any age, though early surgery is recommended. 

In Brazil, the tongue-tie test law (which was signed on June 20^th^, 2014 under Federal Law number 13.002) makes lingual frenum testing compulsory for all newborns. A qualified health-care professional (i.e.: a dentist or speech therapist) must perform the assessment protocol for this test. The clinician should elevate the baby’s tongue to see whether it is tied as well as observe baby’s suckling and crying behaviors. The tongue-tie test should be performed in the maternity within the baby’s first month, for an early diagnosis. This will help prevent breast-feeding problems that may lead to weight loss and unnecessary bottle-feeding. When an abnormally hypertrophic frenum is diagnosed, surgical removal is recommended.^29^

Since the frenum tissue is rather fibrous, local anesthesia should be injected at the base of the frenum. Laser power settings are adjusted to lowest intensity required for that surgery. While some authors recommend 1.6 W^13^ as the ideal setting, in the described case the laser was set to 1.5 W (Fig 7B). After activating the tip with articulating paper, surgery only starts when the laser is horizontally brushed as to detach the frenum, that should be kept stretched for better results (Fig 7C, D, E). Immediately after surgery, a broader range of tongue movement can be perceived (Fig 7F). A week after the procedure, a better shaped tongue tip and an improved range of motion can be observed, as well the presence of granulation tissue, as a result of the secondary intention healing (Fig 7G). 

### Fiberotomy

The tendency for rotated teeth to relapse is one of the main challenges faced after an orthodontic treatment. The slow turnover rate of supracrestal fibers are likely to be responsible for this relapse, which occurs in 48% of cases of patients wearing retainers for as long as 10 years, and is often proportional to the severity of the initial rotation. Apart from the use of orthodontic retainers, only a few additional strategies have been proposed to minimize relapse - the most widely discussed of which is the circumferential supracrestal fiberotomy.^30^ A scalpel is used in the traditional approach, but HILT can be alternatively used while achieving equally satisfactory results.^30^

Erbium mediated lasers are the most recommended devices for fiberotomy (i.e: Er:YAG or ER,Cr:YSGG) for they enable fiber ligaments to reestablish a tissue pattern without inducing superficial necrosis. Although diode laser is another alternative for this procedure, it may cause tissue sloughing, what hinders tissue recovery, extending thus the healing period. So far, we have only found animal studies using diode laser for fiberotomy during the retention period after the orthodontic treatment.^31^ Despite the promising results^31^, further research is required before this procedure can be routinely adopted.

### Other applications 

#### Removal of ceramic brackets

HILT was used to remove ceramic brackets in several studies, with various laser types: Er:YAG,^32^ Nd:YAG,^33^ CO_2_,^34^ and diode laser.^35^ However, questions concerning damages to the dental pulp (due to overheating) as well as to the enamel surface still remain. Further research and more *in vivo* studies are required in order to demonstrate that HILT can be safely used for this purpose. 

#### Enamel etching

The acid-etching concept was proposed by Buonocore^36^ in 1955, and the most common approach consists in applying 37% phosphoric acid to etch the enamel prior to the bonding step. In Orthodontics, HILT may be alternatively used with Er:YAG lasers to promote an appropriate adhesive strength in brackets, with the advantage of avoiding tissue reaction against the acid and gaining better control over the area to be etched. This naturally prevents demineralization from happening in surfaces larger than the bracket area itself.^37^

#### Prevention of white spots

The formation of white spot lesions around braces occur in 50% of treatments. Besides recommending a thorough hygiene required for preventing such lesions, orthodontists can also use fluoride-releasing materials such as Fugi Ortho LC (GC América Inc., Chicago, IL, USA). HILT can also be used as an alternative. According to *in vitro* studies, the CO_2_ laser can change enamel surface around the brackets by reducing carbonate and phosphate contents, what also counts as a caries preventing strategy in these areas.^38^ ^,^ ^39^

#### Recycling of brackets

The recycling of debonded brackets is a cost-effective option that can be performed through different methods in dental offices. Aluminum oxide blasting is a very common technique for cleaning brackets. However the use of Er:YAG laser is an effective process to remove the adhesive from the bracket pad, causing minor impacts to the material and providing brackets with close-to-new bonding strength. 

## DISCUSSION 

**Figure 8:**
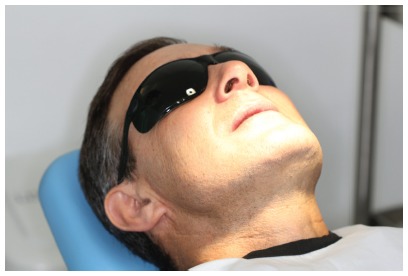
Patient wearing safety glasses during laser therapy.

Although most lasers used in dental practice are relatively user-friendly, precautions should be taken for securing a safe and effective operation. First, everyone subject to laser exposure should wear safety glasses (Fig 8) - that includes dental professionals, assistants, patients, and any other people in the room (patient’s family or friends, for example). The safety glasses should be specifically chosen according to the laser wavelength. Although most lasers emit wavelengths that escape the visible part of the spectrum, their irradiation must not be neglected and caution should be taken. Besides the use of glasses, accidental exposure to laser beams can be avoided by signaling the risk areas with warning signs, limiting access to risk areas, minimizing reflective surfaces, and keeping the equipment under good operation conditions.

Despite the many advantages of using HILT described in this study, some of the laser features should be taken into account before choosing it as the treatment modality. One of the main factors to be considered is that, in spite of favouring healing, laser therapies healing period tend to last longer than conventional dental procedures. The healing period after laser therapy may take 2 to 3 weeks when compared to other procedures that usually take 7 to 10 days.^40^

The use of laser therapies in dental practice is acknowledged in Brazil, with specific training programs available in the market. While any practitioner may operate laser devices, it is recommended that dentists should seek specific training since under-graduate programs seldom include a few hours of laser device practice, if any at all. 

In Brazil, the price of an LLLT equipment ranges between R$ 3,000 to R$ 8,000. HILT equipments tend to be more expensive - a diode laser device is currently priced at R$ 35,000. Considering the investment disbursed by dental professionals to training courses, as well as to purchase and maintain dental equipment, it is recommended that patients be charged specific fees for laser therapy and other laser-aided procedures, even if they are regular ortho patients.

## CONCLUSION 

Despite the many possible indications for Lasers in Orthodontics, it is still underused by Brazilian professionals. However, it is quite likely that this demand will increase over the next years - following the trend in the USA, where laser therapies are more widely used.
